# Role of Terrestrial Wild Birds in Ecology of Influenza A Virus (H5N1)

**DOI:** 10.3201/eid1311.070114

**Published:** 2007-11

**Authors:** Adrianus C.M. Boon, Matthew R. Sandbulte, Patrick Seiler, Richard J. Webby, Thaweesak Songserm, Yi Guan, Robert G. Webster

**Affiliations:** * St Jude Children's Research Hospital, Memphis, Tennessee, USA; † Medicine Kasetsart University, Nakorn Pathom, Thailand; ‡ University of Hong Kong, Hong Kong Special Administrative Region, People’s Republic of China; 1 These authors contributed equally to the study.

**Keywords:** birds, influenza A virus, H5N1, research

## Abstract

House sparrows, European starlings, and Carneux pigeons were inoculated with 4 influenza A (H5N1) viruses isolated from different avian species. We monitored viral replication, death after infection, and transmission to uninfected contact birds of the same species. Sparrows were susceptible to severe infection; 66%–100% of birds died within 4–7 days. High levels of virus were detected from oropharyngeal and cloacal swabs and in organs of deceased sparrows. Inoculation of starlings caused no deaths, despite high levels of virus shedding evident in oropharyngeal swabs. Least susceptible were pigeons, which had no deaths and very low levels of virus in oropharyngeal and cloacal swabs. Transmission to contact birds did not occur frequently: only A/common magpie/Hong Kong/645/2006 virus was shown to transmit to 1 starling. In summary, recent influenza (H5N1) viruses are pathogenic for small terrestrial bird species but the rate of intraspecies transmission in these hosts is very low.

## Abstract 2

Recent viruses are pathogenic for some small terrestrial bird species.

Highly pathogenic avian influenza viruses of subtype H5N1 were identified in Southeast Asia in 1996 and have spread in recent years across broad regions of Eurasia and Africa. These viruses have shown high lethality in chickens and other poultry species (*1*–*3*). Outbreaks of avian influenza, H5N1 subtype and others, have caused massive losses to commercial poultry flocks in recent years (*4*). Direct transmission of H5N1 subtype from infected poultry is thought to be responsible for virtually all of the human influenza (H5N1) infections since 1997. Because of the effects of influenza (H5N1) on human health and agriculture and its potential to mutate and cause a global pandemic, epidemiologic studies of the viruses’ host range and their means of dispersal are urgently needed (*5*).

Highly pathogenic poultry isolates from the 1997 and 2001 influenza (H5N1) outbreaks typically cause few disease signs in experimentally infected ducks (*6*,*7*). These viruses’ low pathogenicity in waterfowl presumably facilitated efficient carriage to the highly susceptible hosts. Some influenza (H5N1) strains isolated during subsequent outbreaks are highly pathogenic in waterfowl (*7*,*8*), and some are shed by infected ducks for prolonged periods (*9*). Together with the commercial transportation of poultry and poultry products, migratory waterfowl are likely to have played a role in the wide dispersal of highly pathogenic influenza (H5N1) viruses.

Land-based wild bird populations may also be vulnerable to lethal influenza (H5N1) infection and could contribute to the spread and interspecies transmission of the viruses. Small terrestrial birds are potentially important hosts in influenza (H5N1) ecology because many of them intermingle freely with wild and domestic populations of waterfowl and poultry. However, data describing their susceptibility to influenza virus (H5N1) infection or their potential to transmit the viruses are limited.

A study investigating the host range of A/chicken/Hong Kong/220/97 showed that it causes lethal infection in budgerigars and finches (*10*). In contrast, the same virus replicated poorly in sparrows, causing no deaths, and, when pigeons were inoculated, replication of this virus was not evident. A more recent chicken influenza (H5N1) isolate (A/chicken/Yamaguchi/7/2004), highly lethal to chickens and quail, also replicates extensively and causes high mortality rates in budgerigars (*11*). Since 2002, influenza (H5N1) viruses have been isolated from dead birds of several wild terrestrial species, including magpie, tree sparrow, pigeon, and large-billed crow (*8*,*12*,*13*). Viruses of a novel influenza (H5N1) genotype were isolated during a survey of live tree sparrows (*Passer montanus)*; these isolates were highly pathogenic to chickens (*14*). Together, these reports indicate that some small, land-based bird species are susceptible to infection, sometimes fatal, with highly pathogenic influenza (H5N1) viruses.

We inoculated sparrows, starlings, and pigeons with several recent highly pathogenic influenza (H5N1) viruses isolated from a variety of avian hosts. The primary aims of the study were to test the susceptibility of different species to infection, investigate the duration and routes of viral shedding from the birds, and assess the possibility of intraspecies viral transmission in these hosts.

## Materials and Methods

### Influenza A Viruses

Four influenza A (H5N1) virus strains were studied, 2 from previously known susceptible hosts (duck and quail) and 2 from previously unknown hosts (common magpie and Japanese white-eye). The A/duck/Thailand/144/2005 (A/DK/TH/144/05) and A/quail/Thailand/551/2005 (A/Q/TH/551/05) viruses were isolated from western Thailand and tested for their pathogenicity in ducks (*15*). The other 2 viruses, A/common magpie/Hong Kong/645/2006 (A/CM/HK/645/06) and A/Japanese white-eye/Hong Kong/1038/2006 (A/JW/HK/1038/06) were provided to us by K.C. Dyrting and C.W.W. Wong (Agriculture, Fisheries and Conservation Department in Hong Kong). They were isolated from dead wild birds collected in January and February 2006 during the heightened Hong Kong territory-wide avian influenza surveillance of dead wild birds that started in October 2005.Upon arrival at St Jude Children’s Research Hospital, the viruses were propagated in 10-day-old embryonated chicken eggs.

### Animal Studies

Wild house sparrows (*Passer domesticus*) and European starlings (*Sturnus vulgaris*), both members of the order Passeriformes, were captured. Six-week-old white Carneux pigeons (*Colomba* spp.), members of the order Columbiformes, were purchased from Palmetto Pigeon Plant (Sumter, SC, USA) and Double T farms (Glenwood, IA, USA). Birds were housed in cages in the St Jude Children’s Research Hospital Animal Biosafety Level 3+ containment facility, food and water were provided ad libitum, and general care was provided as required by the Institutional Animal Care and Use Committee. Before inoculation with virus, oropharyngeal and cloacal swabs were collected to exclude preexisting influenza A virus infection.

Three sparrows and pigeons were inoculated intranasally with 1 million 50% egg infectious doses (EID_50_) in 50 μL or 500 μL phosphate-buffered saline, respectively, for each virus. Because of their limited availability, starlings were inoculated with 3 viruses (1 million EID_50_ in 150 μL), and group sizes were reduced (1 bird for A/DK/TH/144/05, 3 birds for A/CM/HK/645/06, and 2 birds for A/JW/HK/1038/06). One day after inoculation, uninfected contact birds, at a ratio of 1:1 for sparrows and starlings or 2:3 for pigeons, were housed together with inoculated animals to study intraspecies transmission. Birds were monitored daily for death and illness for a 14-day period. After inoculation, oropharyngeal and cloacal swabs were collected on days 2, 4, 6, 8, and 11 for sparrows and starlings and days 3, 5, and 7 for pigeons. Influenza virus was detected by using 10-day-old embryonated chicken eggs as previously described (*7*). EID_50_ virus titers were determined in positive swabs by using the method of Reed and Muench (*15*). The lower limit of quantitation of the assay is 10^0.75^ EID_50_/mL, and average virus titers in organs and swabs were calculated by using the log_10_ value of each sample.

### Serology

Fourteen days after inoculation with virus, serum specimens were collected from inoculated and contact birds, and hemagglutination-inhibition (HI) titers were determined according to standard methods (*16*,*17*) by using chicken erythrocytes and 4 hemagglutinating units of virus. An HI titer >10 suggested a recent influenza virus infection; an HI titer <10 was considered negative.

## Results

### Infection of Different Bird Species with Influenza (H5N1) Virus

The ability of 4 different influenza A (H5N1) viruses to infect and cause disease in house sparrows, European starlings, and white Carneux pigeons was determined. Infection of sparrows caused death in 66%–100% of the infected animals, depending on the inoculated virus (Table 1). The average time to death varied from 4.2 days for A/DK/TH/144/05 to 6.3 days for A/Q/TH/551/05 virus (data not shown). High viral loads were detected in brain and lung tissues of deceased sparrows (Figure, panel C). In contrast, none of the starlings or pigeons died after inoculation with these viruses.

**Table 1:** Influenza A virus titers in oropharyngeal and cloacae swabs of infected birds*

Species	Virus	Mortality rate, %	Virus titer (EID_50_/mL) in swabs from infected animals							
			Day 2			Day 4			Day 6	
			T	C		T	C		T	C
Sparrow	A/DK/TH/144/05	100	2.4	1.7	4.7	4.1	–†	–
	A/Q/TH/551/05	100	1.0	<1		1.5	1.3		3.1	1.0
	A/CM/HK/645/06	66	2.6	0.8		2.3	2.1		1.6	1.1
	A/JW/HK/1038/06	100	2.1	<1		2.7	3.3		–	–
			Day 2			Day 4			Day 6	
			T	C		T	C		T	C
Starling	A/DK/TH/144/05	0	3.8	0.8		3.3	<1		2	<1
	A/Q/TH/551/05	0	ND	ND		ND	ND		ND	ND
	A/CM/HK/645/06	0	3.3	0.8		3.6	1		1.7	1.5
	A/JW/HK/1038/06	0	2.5	1		2	<1		1.8	<1
			Day 3			Day 5			Day 7	
			T	C		T	C		T	C
Pigeon	A/DK/TH/144/05	0	<1	<1		<1	<1		<1	<1
	A/Q/TH/551/05	0	<1	0.5		0.8	<1		<1	<1
	A/CM/HK/645/06	0	1.9	<1		1.4	<1		<1	<1
	A/JW/HK/1038/06‡	0	0.5	<1		0.4	<1		<1	<1

*EID_50_, 50% egg infectious dose; T, oropharyngeal swab; C, cloacal swab; ND, not done. †Birds did not survive the infection. ‡Swabs were taken on days 3, 6, and 10 after infection.

**Figure:**
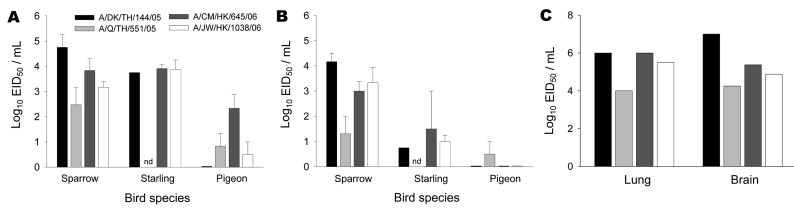
Average peak influenza A virus titers in oropharyngeal (A) and cloacal (B) swabs during the course of influenza (H5N1) infection in 3 terrestrial bird species. C, influenza A virus titers in lungs and brains of deceased sparrows. Data are presented as log_10_ 50% egg infectious doses per milliliter (log_10_ EID_50_/mL). ND, no data available.

Re-isolation of virus from oropharyngeal and cloacal swabs obtained at various time points after inoculation indicated that all the sparrows and starlings were infected by all viruses tested. In contrast, the frequency of virus re-isolation from inoculated pigeons varied widely among viruses. Of the 4 different H5N1 subytpes, A/CM/HK/645/06 demonstrated the broadest host range, infecting not only sparrows and starlings but also all of the inoculated pigeons. The A/DK/TH/144/05 virus, which caused 100% mortality in sparrows within 4.2 days after inoculation, was not re-isolated from inoculated pigeons.

Quantification of the virus titer in the swabs demonstrated that sparrows and starlings shed similar amounts of virus in oropharyngeal swabs. However, virus titers in the cloacal swabs of sparrows were higher than in those obtained from infected starlings (Table 1). Comparison of peak virus titers in oropharyngeal swabs confirmed the similarity in oral shedding between sparrows and starlings. In contrast, peak virus titers in the cloacal swabs of starlings were lower (Figure, panels A and B). The 2005–2006 influenza (H5N1) viruses replicated relatively poorly in pigeons, as shown by average oropharyngeal and cloacal shedding on days 3 and 5 (Table 1) and by peak virus titers in oropharyngeal and cloacal swabs (Figure, panels A and B).

### Intraspecies Transmission of Influenza (H5N1) Viruses

The capacity of current influenza (H5N1) viruses to transmit from infected birds to same-species uninfected birds was assessed for these 4 viruses. No evidence of transmission in sparrows and pigeons was found, as attempts to isolate the virus from contact birds failed (Table 2). Also, no virus-specific antibodies were detected by HI in the contact birds (data not shown). In starlings, transmission of virus to contact birds was observed once for A/CM/HK/645/06 virus, but this was not seen in 2 further experiments.

**Table 2:** Transmission of influenza (H5N1) virus from infected to contact birds of the same species

Species	Influenza A virus			
	A/DK/TH/144/05	A/Q/TH/551/05	A/CM/HK/645/06	A/JW/HK/1038/06
Sparrow	0*	0	0	0
Starling	0	ND†	33	0
Pigeon	0	0	0	0

*Percentage of contact birds from which virus was isolated. †Experiment not done.

## Discussion

The susceptibility of 3 species of wild terrestrial birds to influenza A (H5N1) virus and their ability to transmit to contact birds were assessed. Our studies show that major differences in susceptibility to influenza (H5N1) virus infection exist among these bird species and that, under our conditions, transmission occurred infrequently. Pigeons, starlings, and sparrows were more susceptible to experimental infection with the recent (H5N1) isolates than they were to A/chicken/Hong Kong/220/97 (H5N1) virus (*6*,*10*,*18*). Although drawing conclusions on the basis of a single 1997 isolate is inappropriate, these data are consistent with studies that have demonstrated increased virulence or host range for recent influenza (H5N1) viruses in mammalian species, including mice, ferrets, and domestic and wild cats (*19*–*22*). Whereas a previous study showed that a 2003 chicken influenza (H5N1) isolate can cause severe neurologic disease in pigeons, we observed no signs of disease in influenza (H5N1)–infected pigeons (*23*). Such a difference in pathogenicity between our study and others may be due to subspecies differences or a change in inoculum size.

A critical question concerning these small avian species is whether they can serve as intermediate hosts or reservoirs for influenza (H5N1) viruses and transmit them to poultry and mammals. Sparrows were highly susceptible to influenza (H5N1) infection; however, they did not transmit to sentinel contact birds, despite a relatively low infectious dose (≈500 EID_50_ for A/DK/TH/144/05 virus, data not shown) and the fact that virus was common in drinking water and fecal samples. Although it is possible that the high pathogenicity of these viruses prevented bird-to-bird transmission, the data suggest that this species can act as an intermediate host and potentially transmit to both poultry and mammals but not serve as a reservoir for prolonged shedding of highly pathogenic influenza (H5N1) viruses. In contrast, the characteristics of influenza (H5N1) infection in starlings, i.e., nonfatal with longer-term shedding, suggest that starlings could act as an intermediate host and a reservoir for influenza (H5N1) virus. However, evidence of transmission to contact starlings was limited, which implies that these strains are unsustainable in a starling population. Because pigeons shed only low amounts of virus upon infection and they did not transmit to contact birds, their role in the ecology of influenza (H5N1) virus may be minor.

Our results indicate that there are considerable differences in susceptibility to influenza (H5N1) virus among various small terrestrial wild bird species. The high virulence of several recent isolates in sparrows suggests that this and other populations of small terrestrial birds may have substantial losses during current and future outbreaks. Further mutation of circulating influenza (H5N1) viruses might enhance their adaptation to hosts such as starlings and sparrows, further increasing virulence or allowing these species to become efficient intermediate hosts in the ecology of influenza (H5N1) viruses.
